# Variation in Scheduling and Receipt of Primary Care Follow-up After Hospitalization for COVID-19 in Michigan

**DOI:** 10.1007/s11606-021-07116-6

**Published:** 2021-09-14

**Authors:** Hallie C. Prescott, Bonnie Cheng, Chelsea Abshire, Megan O’Malley, Scott A. Flanders, Renuka Tipirneni, Vineet Chopra

**Affiliations:** 1 Department of Medicine, University of Michigan, Ann Arbor, MI USA; 2 VA Center for Clinical Management Research, Ann Arbor, MI USA; 3 Michigan Value Collaborative, Ann Arbor, MI USA

Patients hospitalized for coronavirus disease (COVID-19) commonly experience new or worsened systemic or cardiopulmonary symptoms, such as fatigue, dyspnea, and weakness^1^. These symptoms may persist for weeks, limit the return to normal activities^2^, and contribute to re-hospitalization^3^. In May 2020, the term “long COVID” was coined to describe the life-altering impacts of COVID-19^4^.

Timely outpatient follow-up has been promoted as a key strategy to reduce readmission and promote recovery after acute illness but does not occur consistently^2,5^. In this study, we examined the variation in primary care scheduling and follow-up across hospitals in Michigan during March–December 2020.

## METHODS

This is an observational cohort study of patients discharged alive from hospitalization for COVID-19 at 38 hospitals in the MI-COVID19 initiative during COVID waves 1 (March–August 2020) and 2 (October–December 2020). Professional abstractors collected data from patient medical records using structured templates^2^. Additionally, patients hospitalized during wave 1 were contacted by telephone to complete a 60-day post-discharge survey that included questions about follow-up care^2^. Measures of interest were scheduling of primary care follow-up prior to discharge and receipt of primary care follow-up within 14 and 30 days of discharge (by telephone, videoconference, or in-person visit). We also examined variation in follow-up practices across hospitals and by hospital type. This study was deemed exempt by the University of Michigan IRB (HUM00179611).

## RESULTS

**Figure 1:**
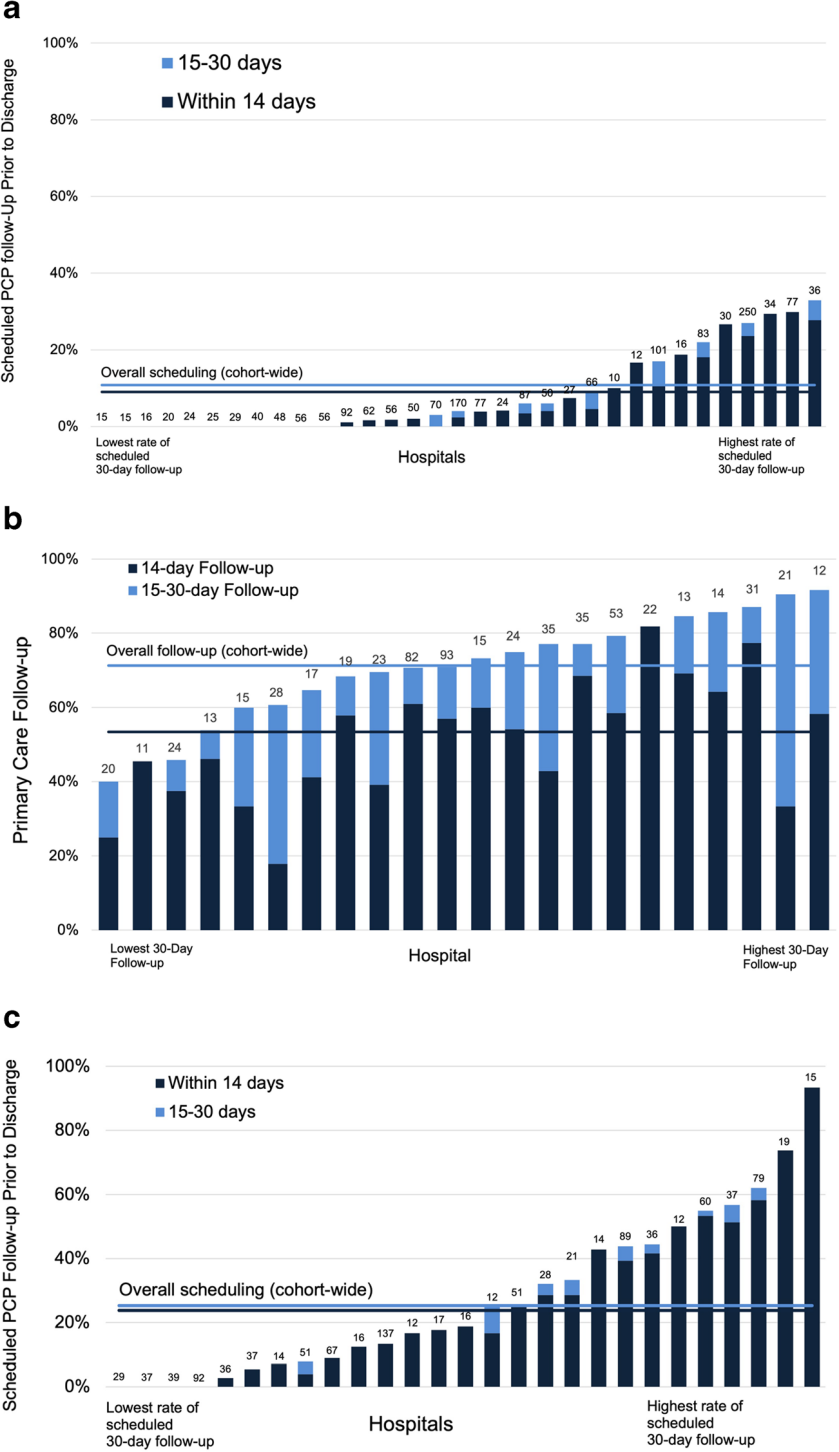
Variation in scheduling and receipt of primary care follow-up across hospitals. Panel **A** Variation in scheduled primary care follow-up across hospitals during wave 1. Primary care follow-up scheduled prior to discharge, to occur within 14 days (navy) and 15–30 days (royal), by the hospital (*N*=33 hospitals; 1824 patients). Each bar represents one hospital; the number of patients per hospital is shown above each bar. Hospitals with fewer than 10 eligible patients are not shown (*N*=4 hospitals; 15 patients). Navy and royal horizontal lines depict the overall proportion of hospitalizations with scheduled 14-day and 30-day follow-up, respectively, prior to discharge. Panel **B** Variation in receipt of primary care follow-up by the hospital during wave 1 Primary care follow-up within 14 days (navy) and 30 days (royal) of hospital discharge, by hospitals (*N* = 22 hospitals; 620 patients). Each bar represents one hospital; the number of patients per hospital is shown above each bar. Hospitals with fewer than 10 patients completing telephone follow-up are not shown (*N*=13 hospitals; 63 patients). Navy and royal horizontal lines depict the overall proportion of hospitalizations with 14-day and 30-day follow-up, respectively. Panel **C** Variation in scheduled primary care follow-up across hospitals during wave 2. Primary care follow-up scheduled prior to discharge to occur within 14 days (navy) and 15–30 days (royal), and at any point (light blue), by the hospital (*N*=27 hospitals; 1063 patients). Each bar represents one hospital; the number of patients per hospital is shown above each bar. Hospitals with fewer than 10 eligible patients are not shown (*N*=4 hospitals; 20 patients). Navy and royal horizontal lines depict the overall proportion of hospitalizations with 14-day and 30-day follow-up, respectively.

Among 1839 patients discharged alive after hospitalization for COVID-19 during wave 1, scheduling of follow-up prior to discharge was rare. Overall, only 9.0% (166/1839) of patients discharge alive were scheduled for 14-day follow-up and 10.8% (198/1839) for 30-day follow-up. Scheduling varied markedly across hospitals (Fig. 1A). In sensitivity analyses excluding patients discharged to post-acute care facilities, findings were similar.

Among 683 patients completing the post-hospitalization survey, 53.4% (365/683) had primary care follow-up within 14 days, and 71.3% (487/683) within 30 days of discharge. Follow-up varied across hospitals, ranging from 17.9 to 81.8% for 14-day and 40.0 to 91.7% for 30-day follow-up (Fig. 1B). Receipt of 14-day follow-up was more common among patients scheduled for 14-day follow-up prior to discharge (66.7% (44/66) vs 52.0% (321/617), *p*=0.023), but receipt of 30-day follow-up did not differ by scheduling (80.0% (60/75) vs 70.2% (427/608), *p*=0.078).

During wave 2, scheduling of primary care follow-up increased but still occurred for only a minority of patients. A total of 23.8% (258/1083) had 14-day and 25.3% (274/1083) 30-day follow-up scheduled prior to discharge. Variation in scheduling persisted across hospitals (Fig. 1C).

Across waves 1 and 2, scheduled follow-up was higher among patients discharged from non-profit versus for-profit hospitals but similar for patients discharged from teaching versus non-teaching hospitals (Table 1).

**Table 1:** Proportion of Patients with Primary Care Follow-up Scheduled Prior to Discharge by Wave and Hospital Type

	Number		
		Within 14 days	Within 30 days
Total wave 1 live discharges	1839	9.0%	10.8%
Discharged from a teaching hospital	1667	8.9%	10.5%
Discharged from non-teaching hospital	172	10.5%	13.4%
Discharged from non-profit hospital	1738	9.4%	11.2%
Discharged from proprietary hospital	101	2.0%	4.0%
Total wave 2 live discharges	1083	23.8%	25.3%
Discharged from a teaching hospital	975	24.2%	25.6%
Discharged from non-teaching hospital	108	20.4%	22.2%
Discharged from non-profit hospital	1008	25.1%	26.7%
Discharged from proprietary hospital	75	6.7%	6.7%

Data on hospital ownership status were obtained from data.medicare.gov; Hospital General Information. Retrieved from 06/03/2020 from https://data.medicare.gov/widgets/xubh-q36u

## DISCUSSION

In this multi-hospital cohort of patients hospitalized for COVID-19 in Michigan, there was marked variation in primary care follow-up. Two-week follow-up varied more than 4-fold across hospitals during wave 1, and fewer than 10% of the patients were scheduled for a 14-day follow-up prior to discharge. During wave 2, 25% were scheduled for a 14-day follow-up prior to discharge, but hospital-level variation persisted. At the extremes, four hospitals scheduled no follow-up, while one hospital scheduled follow-up for >90% of patients.

Outpatient follow-up visits are a key opportunity to screen for, acknowledge, and address the multi-faceted symptoms after COVID-19^1^ and other acute illnesses. While the long-term sequelae of COVID-19 were not well-known until wave 2, 2-week follow-up is encouraged by the Centers for Medicare and Medicaid Services and may accelerate evaluation and management of problematic symptoms (e.g., functional impairments) after hospitalization. Thus, while our data are specific to COVID-19 hospitalization, they also reflect a broader issue of care fragmentation and poor transition from hospital to primary care^6^.

Limitations of this study include ascertainment of primary care scheduling and visits from the medical record and patients’ self-report, respectively. Furthermore, receipt of primary care follow-up was measured among a subset of patients who completed a telephone survey. The response rate to this survey was low (42.7%), but demographics and illness severity were similar among respondents versus non-respondents.

This study highlights the incomplete scheduling and receipt of primary care follow-up after COVID-19 hospitalization, as well as marked variation in scheduling practices across hospitals. The reasons for this hospital variation are unclear and warrant further exploration. Enhanced policies and programs to facilitate post-hospitalization follow-up appear necessary.
